# P-2009. COVID-19 in Children with Hematological/Oncological Diagnoses – Exploring Reasons for Better Clinical Outcomes

**DOI:** 10.1093/ofid/ofae631.2166

**Published:** 2025-01-29

**Authors:** Swetha Pinninti, Connie Trieu, Barbora Knoppova, Sunil Pati, Avangelos Barley, Misty Latting, Sama Halima, Alexis Ridings, Sydney Poulson, Kimberly Whelan, Christina Bemrich-Stolz, William Britt, Suresh Boppana

**Affiliations:** University of Alabama at Birmingham, Birmingham, Alabama; Daffodil Pediatrics, Atlanta, Georgia; University of Alabama at Birmingham, Birmingham, Alabama; University of Alabama at Birmingham, Birmingham, Alabama; University of Alabama at Birmingham, Birmingham, Alabama; University of Alabama at Birmingham, Birmingham, Alabama; University of Alabama at Birmingham, Birmingham, Alabama; University of Alabama at Birmingham, Birmingham, Alabama; University of Alabama at Birmingham, Birmingham, Alabama; University of Alabama at Birmingham, Birmingham, Alabama; University of Alabama at Birmingham, Birmingham, Alabama; Division of Pediatric Infectious Diseases, University of Alabama at Birmingham, Birmingham, Alabama; University of Alabama at Birmingham, Birmingham, Alabama

## Abstract

**Background:**

The majority of COVID-19 infections in children, including in those with underlying hematological and oncological (Heme-Onc) diagnoses are mild with good outcome compared to adults. Postulated reasons include cross-protection from immunity against seasonal coronaviruses (HCoV’s), subdued inflammatory responses in children than in adults and differences in humoral and T-cell responses. The objectives of this study are to compare antibody responses against HCoV’s, anti-SARS-CoV-2 humoral, T-cell, and inflammatory profiles between Heme-Onc children with COVID-19 and control groups.

**Figure:**
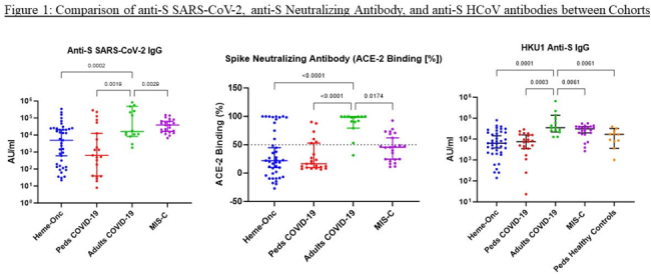

**Methods:**

Binding IgG antibodies against Spike (S) of SARS-CoV-2, four HCoV’s (HKU1, OC43, NL63 and 229E), quantitative levels of 21 cytokines and 9 chemokines (multiplexed solid-phase electrochemiluminescence assay by Meso Scale Diagnostics/MSD) were compared among five cohorts: 1) children with Heme-Onc diagnoses and COVID-19 (n = 41) 2) healthy children with COVID-19 (n = 21) 3) adults with COVID-19 (n = 14) 4) children with multisystem inflammatory syndrome (MIS-C) [n = 23] and 5) healthy children without COVID-19 (n = 12). Additionally, T-cell responses against S and nucleocapsid (N) proteins of SARS-CoV-2 was assessed using ELISpot in Heme-Onc children a group of healthy children COVID-19.

**Results:**

Anti-S antibodies and neutralizing antibodies against SARS-CoV-2 and HCoV were significantly lower in children in Heme-Onc and healthy children with COVID-19 compared to adults with COVID-19 (Figure 1). The concentration of eight pro-inflammatory cytokines and chemokines were significantly lower in Heme-Onc and healthy children compared to adults with COVID-19 and children with MIS-C. The responder T-cell frequency varied between 0 and 860/100,000 PBMC at study enrollment in children with Heme-Onc diagnoses.

**Conclusion:**

In this single-center study, cross-protection from HCoV antibodies is an unlikely mechanism to explain mild COVID-19 in children, including in those with underlying hematological and oncological diagnoses. Subdued inflammatory responses to SARS-CoV-2 could be one of the factors for the mild COVID-19 disease phenotype in children.

**Disclosures:**

Swetha Pinninti, MD, Moderna: Grant/Research Support|Pfizer: Grant/Research Support Suresh Boppana, MD, GSK: Advisor/Consultant|Merck: Grant/Research Support|Pfizer: Grant/Research Support

